# Increases in RPE Rating Predict Fatigue Accumulation Without Changes in Heart Rate Zone Distribution After 4-Week Low-Intensity High-Volume Training Period in High-Level Rowers

**DOI:** 10.3389/fphys.2021.735565

**Published:** 2021-09-16

**Authors:** Rasmus Pind, Peter Hofmann, Evelin Mäestu, Eno Vahtra, Priit Purge, Jarek Mäestu

**Affiliations:** ^1^Institute of Sport Sciences and Physiotherapy, University of Tartu, Tartu, Estonia; ^2^Institute of Human Movement Science, Sport and Health, University of Graz, Graz, Austria

**Keywords:** training monitoring, intensity, duration, internal load, external load, exercise prescription, session RPE

## Abstract

**Purpose:** The aim of this study was to investigate the interaction of training load quantification using heart rate (HR) and rating of perceived exertion (RPE)-based methodology, and the relationship between internal training load parameters and subjective training status (*Fatigue*) in high-level rowers during volume increased low-intensity training period.

**Methods:** Training data from 19 high-level rowers (age 23.5 ± 5.9 years; maximal oxygen uptake 58.9 ± 5.8 ml·min^−1^·kg^−1^) were collected during a 4-week volume increased training period. All individual training sessions were analyzed to quantify training intensity distribution based on the HR time-in-zone method (i.e., HR Z1, HR Z2, and HR Z3) determined by the first and second ventilatory thresholds (VT1/VT2). Internal training load was calculated using session RPE (sRPE) to categorize training load by effort (i.e., sRPE1, sRPE2, and sRPE3). The Recovery-Stress Questionnaire for Athletes (RESTQ-Sport) questionnaire was implemented after every week of the study period.

**Results:** No differences were found between the respective HR and effort-based zone distributions during the baseline week (*p* > 0.05). Compared to HR Z1, sRPE1 was significantly lower in weeks 2–4 (*p* < 0.05), while sRPE2 was higher in weeks 2–3 compared to HR Z2 (*p* < 0.05) and, in week 4, the tendency (*p* = 0.06) of the higher amount of sRPE3 compared to HR Z3 was found. There were significant increases in RESTQ-Sport stress scales and decreases in recovery scales mostly during weeks 3 and 4. Increases in the *Fatigue* scale were associated with the amounts of sRPE2 and sRPE3 (*p* = 0.011 and *p* = 0.008, respectively), while no associations with *Fatigue* were found for HR-based session quantification with internal or external training load variables.

**Conclusion:** During a low-intensity 4-week training period with increasing volume, RPE-based training quantification indicated a shift toward the harder rating of sessions with unchanged HR zone distributions. *Moderate* and *Hard* rated sessions were related to increases in *Fatigue*. Session rating of perceived exertion and effort-based training load could be practical measures in combination with HR to monitor adaptation during increased volume, low-intensity training period in endurance athletes.

## Introduction

Athletes frequently use manipulations in training load (i.e., intensity, duration, and frequency) to stimulate training adaptation. For example, it has been clearly demonstrated in rowers that low-intensity training kilometers are positively related to success in championships (Hagerman and Staron, 1982; Steinacker, 1993; Mäestu et al., 2005), and therefore periods of low-intensity and high-volume training are frequently used during preparation to optimize performance. Such increases in low-intensity training may reach up to 50% of the regular training volume (Rämson et al., 2008; Buchheit et al., 2013; Comotto et al., 2015; Thornton et al., 2017). However, the risk of overreaching/overtraining increases with increased training volume and particularly with monotonous training (Fry et al., 1992; Lehmann et al., 1992, 1993; Meeusen et al., 2013). Therefore, the biggest challenge in the training and monitoring process is to determine the time point where adaptive training might turn maladaptive. However, due to delayed effects and several interdependencies, it is a complex and complicated process and difficult to measure. By the time an underlying problem has been confirmed in the laboratory, the competitive results of athletes may already be compromised (Meeusen et al., 2013). Therefore, with the aim to optimize performance in the training cycles, training sessions need careful planning and monitoring to improve performance and to avoid nonfunctional overreaching (a process of increased training load in combination with insufficient recovery) (Meeusen et al., 2013; ten Haaf et al., 2017), or even overtraining syndrome.

Training load can be described by two different concepts, namely, external (i.e., training time, covered distance, and lifted weight) and internal (i.e., heart rate (HR), oxygen uptake, and generated power). Internal training load is the actual response of the body to the applied external load. To describe internal load, the organization of the training intensity continuum into specific zones is common, most frequently defined by HR zones (Seiler and Kjerland, 2006), which can further be applied to calculate training load by using, for example, the Training Impulse (TRIMP) method. Lucia et al. (2003) developed a method with the use of the first and second ventilatory thresholds (VT1/VT2) as physiological landmarks to define three HR zones and a subsequent weighing factor to calculate training load. However, such an HR-based time-in-zone approach might underestimate the time spent working at high-intensity (due to HR lag time during intervals) or the HR drift over the course of a longer workout (Seiler and Kjerland, 2006). An additional practical method to measure internal load is the use of the training session rating of perceived exertion (sRPE), which reflects the subjective response of an individual to training load (Foster et al., 2001), taking into account both the duration and intensity components. The sRPE method is a simple and valid method (Foster, 1998; Foster et al., 2001), and has already been applied in different endurance sports (Foster et al., 2001; Wallace et al., 2014; Roos et al., 2018; Sanders et al., 2018) including rowing (Tran et al., 2015) and has shown high reliability with different objective HR-based methods (Seiler and Kjerland, 2006; Rodríguez-Marroyo et al., 2012; Lupo et al., 2014; García-Ramos et al., 2015). In addition to general “overall internal load” parameters of the training session, previous studies have also used rating of perceived exertion (RPE) as effort-based quantification to distinguish between easy or hard training sessions based on RPE value using the same VT1/VT2 anchor points as for Lucia TRIMP (Seiler and Kjerland, 2006; Wallace et al., 2009). Accordingly, we can further calculate training load as *Easy, Moderate*, or *Hard* sessions (Pind et al., 2019). In accordance with the suggestion that training load quantification methods should have high dose–response validity with the changes in fitness and/or performance (Sanders et al., 2017), it was recently presented that training load from hard sessions was related to VO_2max_ improvements in swimmers preparation for competitions (Pind et al., 2019).

Although RPE or sRPE have been mostly considered measures of exercise intensity (Foster et al., 2001), recent studies suggest that sRPE could be affected by other factors, i.e., duration of the session or fatigue. The recent experimental study indicated that during 30 min constant running exercise, RPE values were higher compared to similar intensities during a 15-min run (Jesus et al., 2021). However, the effect was seen for intensities described as moderate or hard and not for low-intensity exercise. Additionally, Fusco et al. (2020a) indicated that during extensive interval swimming session (blood lactate concentration around 6 mmol L^−1^), RPE increased constantly throughout the session if additional interval blocks were added. Furthermore, it was found that using constant high-intensity sessions, HR and RPE values indicated a relatively constant pattern over the 2-week period (Fusco et al., 2020b). However, there were significant differences between RPE values at the end of the training period compared to the reference training at the beginning of the study. Using the data from the cycling Grand Tours, Sanders et al. (2017) proposed that changes in the ratios of intensity and load measures (including measures of HR and RPE) could reflect increasing fatigue that might not be well detected by analyzing solitary intensity/load measures. Therefore, the integration of RPE and HR data could provide additional information about the fatigue status or overtraining risk of athletes. However, there is limited information of the use of RPE and HR integration during a high-volume, low-intensity training cycle that is commonly used in rowing during the preparatory period to obtain subjective fatigue development.

As indicated in previous studies (Meeusen et al., 2013; Halson, 2014), the subjective or psychometric instruments are sensitive in terms of changes either for training load or for performance. The advantage is that they are relatively simple and inexpensive to determine the status of an athlete and his/her response to the training session or training cycles (Steinacker et al., 2000). The Recovery-Stress Questionnaire for Athletes (RESTQ-Sport) was developed to measure the frequency of current stress along with the frequency of recovery-associated activities (Kellmann and Kallus, 2001) and has been shown effective to monitor the training status of rowers (Kellmann and Günther, 2000; Kellmann et al., 2001; Mäestu et al., 2006). Recent studies relating changes in training load and psychometric instruments exclusively studied the manipulation of external load during high-load training periods (Steinacker et al., 2000; Jürimäe et al., 2004; González-Boto et al., 2008; Scott and Lovell, 2018) and suggested that changes in the external training load are reflected by changes in the RESTQ-Sport scales (Mäestu et al., 2006; González-Boto et al., 2008). However, the interaction between subjective instruments and changes in the internal training load has been less focused (Buchheit et al., 2013; Comotto et al., 2015; Collette et al., 2018). A recent study with junior-elite triathletes (Comotto et al., 2015) evaluated the individual responses to training by monitoring sRPE and Profile of Mood States. These authors suggested that monitoring of mood and perceived exertion during periods of heavy training may help individualize training to prevent overtraining during training camps. Therefore, the purpose of this study was to investigate the interaction of training load quantification by HR and RPE, and the relationship between internal training load parameters and subjective training status (*Fatigue*) during a 4-week training cycle with increasing training volume in high-level rowers.

## Materials and Methods

### Subjects

Participants of this study were 27 elite rowers of the Estonian National Rowing Team. Depending on the age group, they were the members of the National U-19, U-23, or Senior A-Team. During the study period, eight subjects were excluded from the analyses, due to the following reasons: (i) having provided <95% of training data or (ii) having missed more than 5% of the training sessions due to sickness or other reasons. Therefore, the number of participants included in the final analyses was 19 [age 23.5 ± 5.9 years; height 1.87 ± 0.07 m; body mass 87.0 ± 11.0 kg; body mass index 24.7 ± 1.9 kg·m^−2^; and maximal oxygen uptake (VO_2max_) 58.9 ± 5.8 ml·min^−1^·kg^−1^].

### Experimental Procedures (Design)

All subjects participated in a 4-week training camp at the end of the winter preparatory period from March to April. An introductory meeting to describe the details of the study was organized a week before the training camp, during which the purpose, study design, and measurements were explained. Before the first measurements, participants, or their legal guardians for those under the age of 18, gave the written informed consent to participate in the study. The study was approved by the institutional ethical committee conducted in accordance with the Declaration of Helsinki (7th edition) (Hellmann et al., 2014).

During the first visit to the lab, height and body mass of the participants were measured. Second, an incremental exercise test until volitional exhaustion was performed on a rowing ergometer (Concept II, Model B, Morrisville, VT, USA) with an initial workload of 40 Watts (W) and increments of 20 W every minute (Hofmann et al., 2007). Expired gases and HR were continuously measured using a portable metabolic device (Metamax 3B, Cortex Biophysik GmbH, Leipzig, Germany) to determine performance parameters. VO_2max_ was defined as the highest average VO_2_ during a 30-s period. To ensure reaching the maximal effort, the following criteria were used: failure to increase VO_2_ despite an increase in work rate or respiratory exchange ratio exceeding 1.1. The VT1 and the VT2 were determined as shown previously (Hofmann et al., 2007). In brief, VT1 was determined as the first increase in ventilation (VE) accompanied by an increase in the equivalent for oxygen uptake (VE/VO_2_) without an increase in the equivalent for carbon dioxide output (VE/VCO_2_). VT2 was determined as the second distinct increase in VE accompanied by an increase in both VE/VO_2_ and VE/VCO_2_. All determinations were performed within defined regions of interest such as between the first workload and 65% of maximal performance (P_max_; W) for VT1 and between VT1 and P_max_ for VT2. Determinations were performed by visual inspection from two independent and experienced researchers. If there was disagreement between the two observers, a third reviewer was used. HR was measured continuously and registered every 5s *via* chest strap telemetry (Polar Electro. Kempele, Finland). Individual VT1 and VT2-related target HR zones were used in athletes to quantify their training intensity during the training as follows: HR Z1 (the time with HR values lower or equal to VT1); HR Z2 (the time with HR values between VT1 and/or equal to VT2); and HR Z3 (the time with HR values above VT2).

### External and Internal Training Load

The first week of the study was characterized as the baseline week, for the athletes to adapt to the training environment, without an increase in training load compared to previous weeks. During the next 2 weeks, training volume was increased by about 50% and the fourth week was organized as a recovery microcycle, where training volume was planned to decrease to about 40% of the high-volume weeks. HR was recorded using HR monitors (Polar M400, Polar OY, Kempele, Finland) during every single training session. All individual training sessions were downloaded to quantify training intensity distribution based on the time-in-zone method using the predetermined individual HR zones. In addition to HR data, athletes were instructed how to use an online training log and sports coaching software (Sportlyzer, Tartu, Estonia) for recording all training sessions during 4 weeks, including the mode of training, duration of each session, and how to record RPE on a 10-point scale. Participants were all familiar with the RPE scale before the study, as this has already been part of their previous training routine. The RPE was recorded 30 min after the end of each training session with the value from 0 to 10, answering the question: “How hard was your workout?” Internal load of each session was determined by the sRPE method—RPE multiplied by the duration (min) of the session (Foster et al., 2001). The 10-point RPE responses were further used to categorize training sessions by effort to describe the entire session as either *Easy* (training sessions rated as 4 or less on a 10-point RPE scale; RPE ≤ 4, sRPE1), *Moderate* (training sessions rated as 5 or 6; sRPE2), or *Hard* (training sessions rated as 7 or higher; sRPE3) using VT1 and VT2 cutoff points as previously indicated (Seiler and Kjerland, 2006). HR and RPE from strength sessions were not included in the analysis.

### RESTQ-Sport Questionnaire

The Recovery-Stress Questionnaire for Athletes is a psychometric instrument that can be used to classify individuals overreached (Kellmann and Günther, 2000) and it consists of 77 items (19 scales with 4 items each plus 1 warm-up item). A Likert-type scale is used with values ranging from 0 (never) to 6 (always), indicating how often the respondent participated in various activities during the past 3 days/nights. The first seven scales cover different aspects of subjective strain as well as the resulting consequences (Table 1). The next five scales are the basic scales for the recovery area *with Success* as the only resulting recovery-oriented scale concerned with performance in general, but not in a sport-specific context (Kellmann and Günther, 2000). Sport-specific details of stress and recovery are examined in scales 13–19 (Kellmann and Günther, 2000; Kellmann and Kallus, 2001). The Estonian version of the questionnaire (Mäestu et al., 2006) was implemented every Monday starting after the baseline week of the training camp. Therefore, it was implemented four times—after every week (first, second, third, and fourth) of the training camp. Participants completed the questionnaire always after breakfast to keep the time schedule comparable.

**Table 1:** Average scores (mean ± SD) of the Recovery-Stress Questionnaire for Athletes (RESTQ-Sport) scales during the 4-week training period.

**Scale**	**Baseline week**	**Week 2**	**Week 3**	**Week 4**	***P*-value**
**General stress**					
1. General stress	1.01 ± 0.93	1.03 ± 1.08	1.25 ± 1.03	1.97 ± 1.68	ns
2. Emotional stress	1.21 ± 0.90	1.13 ± 1.10	1.28 ± 0.88	2.09 ± 1.58	ns
3. Social stress	1.29 ± 0.82	1.11 ± 0.94	1.33 ± 0.91	**2.25** **± 1.38**^**b**^	*p* < 0.05
4. Conflicts/pressure	1.65 ± 0.90	1.61 ± 0.87	1.93 ± 0.71	**2.59** **± 1.19**^**b**^	*p* < 0.05
5. Fatigue	1.74 ± 1.19	1.61 ± 1.11	**2.07** **± 1.23**^**b**^	**2.69** **± 1.50**^**b**^	*p* < 0.05
6. Lack of energy	1.81 ± 0.82	1.53 ± 0.69	1.70 ± 0.73	1.75 ± 0.53	ns
7. Physical complaints	1.44 ± 0.75	1.52 ± 0.83	1.68 ± 0.64	**1.91** **± 0.58**^**a**^	*p* < 0.05
**General recovery**					
8. Success	3.11 ± 0.79	2.75 ± 1.25	2.55 ± 0.92	2.75 ± 0.88	ns
9. Social recovery	3.57 ± 1.36	3.42 ± 1.35	3.47 ± 1.31	2.50 ± 1.20	ns
10. Physical recovery	3.18 ± 1.18	3.08 ± 1.08	2.83 ± 1.07	2.34 ± 1.18	ns
11. General well-being	3.96 ± 1.32	3.94 ± 1.31	3.93 ± 1.27	**2.69** **± 1.27**^**a**^	*p* < 0.05
12. Sleep quality	4.00 ± 1.08	**4.53** **± 0.90**^**a**^	4.15 ± 1.13	3.31 ± 1.46	*p* < 0.05
**Sport stress**					
13. Disturbed breaks	1.47 ± 0.82	1.09 ± 0.50	**1.63** **± 0.93**^**b**^	**1.94** **± 0.94**^**b**^	*p* < 0.05
14. Emotional exhaustion	1.07 ± 1.36	1.03 ± 1.14	1.30 ± 1.31	1.69 ± 1.93	ns
15. Injury	2.13 ± 0.98	2.19 ± 1.03	**2.03** **± 0.92**^**b**^	1.63 ± 0.82	*p* < 0.05
**Sport recovery**					
16. Being in shape	3.51 ± 1.11	3.39 ± 1.24	3.00 ± 1.09	2.84 ± 0.88	ns
17. Personal accomplishments	3.19 ± 0.99	2.88 ± 1.14	**2.50** **± 1.29**^a^	**2.22** **± 1.48**^a^	*p* < 0.05
18. Self-efficacy	3.44 ± 0.76	3.41 ± 0.98	3.03 ± 1.00	3.25 ± 0.96	ns
19. Self-regulation	2.31 ± 0.83	2.47 ± 0.88	2.37 ± 0.94	**3.44** **± 1.24**^**b**^	*p* < 0.05

a *p < 0.05 is significantly different from baseline week*.

b *p < 0.05 is significantly different from week 2. ns, non-significant. The statistical differences (p < 0.05) are shown in bold*.

### Statistical Analysis

Descriptive statistics of the subjects are presented as mean values and SDs. Before analyses, the assumption of normality was assessed by using the Shapiro–Wilk test. The one-way repeated measures of ANOVA was used to test for mean differences between the four time points for measured parameters with Bonferroni adjustment. The two-way repeated measures of ANOVA was used to test the interaction between the time and quantification method. In addition, differences between HR and sRPE distribution (%) at different time points (i.e., HR Z1, HR Z2, and HR Z3) and training loads (i.e., sRPE1, sRPE2, and sRPE3) were assessed with a paired-sample *t*-test. An automatic linear modeling was carried out to explore the main predictors of the *Fatigue* score of the RESTQ questionnaire. The variables inserted into the model as independent variables were training volume, distance, total training load, sRPE1, sRPE2, sRPE3, HR Z1, HR Z2, and HR Z3.

## Results

**Figure 1:**
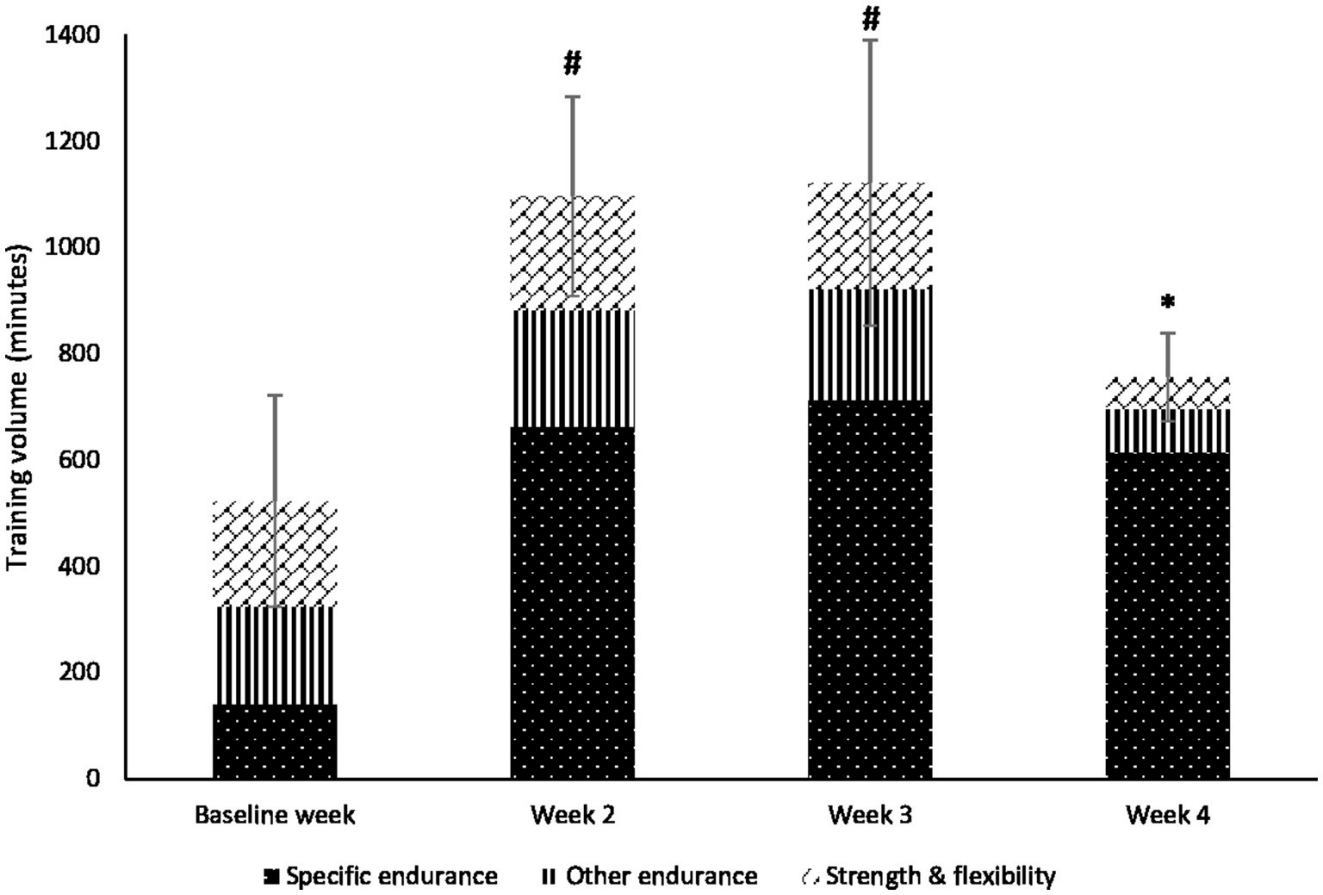
Total training volume (minutes) and the volumes of each training type during the 4-week training period. Specific endurance includes indoor rowing (ergometer) and outdoor rowing. Other endurance includes all types of other endurance sports—mostly running and cycling; ^*^Significantly different total training volumes from baseline week (*p* < 0.05); #Significantly different total training volumes from baseline week to week 4 (*p* < 0.05).

Total training volume during the 4-week training period was 58.3 ± 8.8 h (Figure 1). Training volume significantly changed over time [*F*_(3,54)_ = 57.927, *p* < 0.001]. Furthermore, training volume increased by 52% from 522.5 ± 198.5 min in the baseline week to 1096.6 ± 187.9 min in week 2 (*p* < 0.001). The highest training volume was achieved during week 3 with 1121.7 ± 268.6 min of training and then decreased (*p* < 0.001) to 755.8 ± 82.7 min during the recovery week as planned for the four microcycles. The increase in training volume was the result of the number of the training sessions that changed significantly [*F*_(3,54)_ = 26.703; *p* < 0.001] and resulted in an increase from baseline (9.1 ± 2.7) to weeks 2 and 3 (15.8 ± 4.4 and 15.9 ± 4.7, respectively). There was a significant change in the average length of training sessions over four time points [*F*_(3,54)_ = 5.460, *p* = 0.006], with a significant increase from 64.0 ± 21.3 min at baseline to approximately 75.5 min both in weeks 2 and 3 (*p* < 0.05) and a significant decrease to 61.7 ± 12.8 min in week 4 (*p* < 0.05). No changes were found for the volume of strength and flexibility training during weeks 1–3, but a significant decrease in week 4 (*p* < 0.05), compared to all previous weeks, was found.

**Figure 2:**
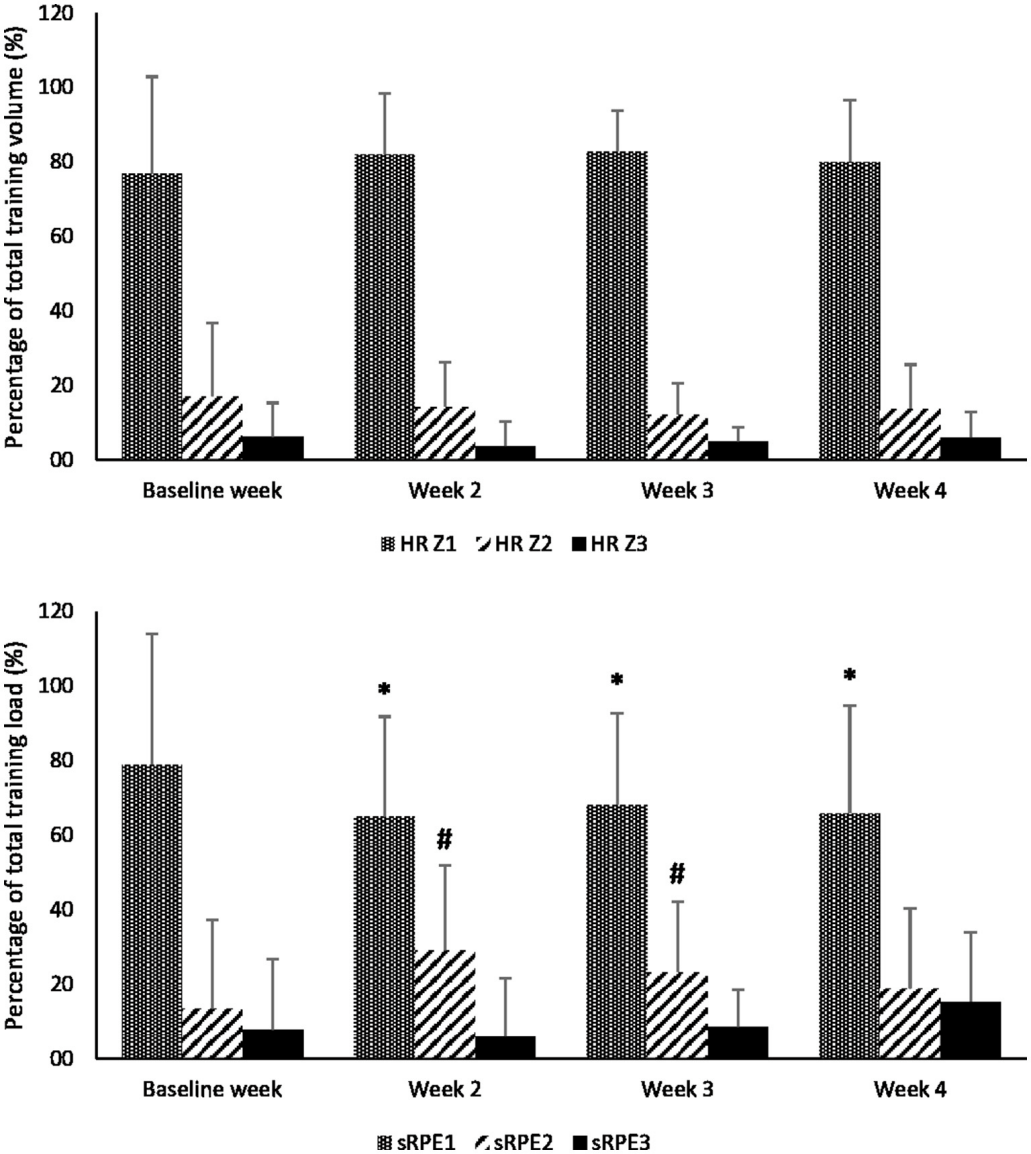
Differences between the percentages (%) in heart rate zone distributions (i.e., HR Z1, HR Z2, and HR Z3; upper panel) and the respective effort-based zone distributions (i.e., sRPE1, sRPE2, and sRPE3; lower panel) during the 4-week training period; ^*^Significantly different from HR Z1 *p* < 0.05; #Significantly different from HR Z2 (*p* < 0.05).

Accumulated load (sRPE) during the 4-week training period was 12388.22 ± 3190.12 arbitrary units (AUs). There was an overall change in weekly internal training load over the four time points [*F*_(3,54)_ = 42.711, *p* < 0.001]. In addition, the weekly internal load was significantly different (*p* < 0.05) between all training weeks except between the second and third weeks (*p* = 0.845). Training session categorization according to HR (i.e., Z1, Z2, and Z3) and effort-based methods (i.e., sRPE1, sRPE2, and sRPE3) can be found in Figure 2. About 80% of the sessions was performed within HR Z1 below VT1 and only about 5% of sessions in HR Z3. Although the HR distribution within the zones did not change during the 4 weeks, the subjective rating of internal load (sRPE) presented a significant shift from low to moderate and high load (subjective strain) during weeks 2 and 3, which resulted in a significant change in sRPE2 over the study period [*F*_(3,54)_ = 2.881; *p* = 0.044]. No other changes over time were found for different zones.

There was significant time × quantification method interaction in HR Z1 vs. sRPE1 [*F*_(3,51)_ = 3.970; *p* = 0.013] and in HR Z2 vs. sRPE2 [*F*_(3,51)_ = 0.906; *p* = 0.045]. The respective distributions of the three zones were not different in the baseline week if comparing the HR and RPE quantification methods (*p* > 0.05). However, the distribution of sRPE1 was lower compared to HR Z1 in weeks 2–4 (*p* < 0.05), and sRPE2 was higher in weeks 2–3 compared to HR Z2 (*p* < 0.05). No time × quantification method interaction in HR Z3 vs. sRPE3 [*F*_(3,51)_ = 0.906; *p* = 0.445] was found. However, in week 4, there was a tendency (*p* = 0.06) for a higher sRPE3 compared to HR Z3.

If applying a zone distribution according to an 80:20 principle (Seiler, 2010), the proportion of sRPE2+sRPE3 training did not change over time [*F*_(3,54)_ = 1,379, *p* = 0.260], but a tendency (*p* = 0.080) for an increase was found between baseline and week 4 (from 21.0 to 34.1%, respectively).

Significant increases (*p* < 0.05) during the 4 weeks were found in the stress scales, namely, *Social Stress, Conflicts/Pressure, Fatigue, Physical Complaints*, and *Disturbed Breaks and Injury* (Table 1), while recovery scale changes (*p* < 0.05) were seen between the weeks in the scales, namely, *General Well-Being, Sleep Quality, Personal Accomplishments*, and *Self-Regulation*. Most of the changes in the scales appeared in the final 2 weeks (week 3 and week 4) of the study. During the final week, significant increases were mostly found in the stress scales, namely, *Social Stress* (*p* = 0.046, week 2 and week 4); *Conflicts/Pressure* (*p* = 0.025, week 2 and week 4); *Fatigue* (*p* = 0.046, week 2 and week 4); *Physical Complaints* (*p* = 0.011, baseline week and week 4); and *Disturbed Breaks* (*p* = 0.021, week 2 and week 4). In contrast, significant decreases were found in the following recovery scales during the final week of the study period: *General Well-Being* (*p* = 0.048, baseline week and week 4) and *Self-Regulation* (*p* = 0.022, week 2 and week 4).

According to the automatic linear modeling, three important predictors of the RESTQ-Sport scale *Fatigue*, namely, overall training load (sRPE), sRPE3, and sRPE2 (Table 2) were found. sRPE3 and sRPE2 were associated positively with *Fatigue* while negative associations were found for sRPE (Table 2). No relationships were found for HR-based quantification zones.

**Table 2:** An automatic linear modeling to indicate the main predictors of *Fatigue* scale levels.

**Model term**	**Coefficients**	**SE**	***p*** **value**	**Importance**
Intercept	2.724	0.470	<0.001	
Training volume (min)	0.003	0.002	0.121	0.078
Training distance (km)	0.001	0.004	0.784	0.002
sRPE (AU)	−0.002	0.001	**0.007**	0.251
sRPE1 (AU)	0.001	0.001	0.279	0.037
sRPE2 (AU)	0.002	0.001	**0.011**	0.219
sRPE3 (AU)	0.002	0.001	**0.008**	0.239
HR Z1 (min)	−0.002	0.001	0.193	0.054
HR Z2 (min)	0.001	0.003	0.735	0.004
HR Z3 (min)	−0.015	0.008	0.089	0.116

*SE, standard error; AU, arbitrary unit; sRPE, session rating of perceived exertion; sRPE1, internal load of training sessions rated as 4 or less on a 10-point RPE scale; sRPE2, internal load of training sessions rated as 5 or 6; sRPE3, internal load of training sessions rated as 7 or higher; HR Z1, heart rate values below ventilatory threshold 1; HR Z2, heart rate values between ventilatory thresholds 1 and 2; HR Z3, heart rate values above ventilatory threshold 2. The statistical differences (p < 0.05) are shown in bold*.

## Discussion

The purpose of this study was to investigate the interaction of HR and RPE-based training quantification methods and subjective parameters to similarly prescribed external load and their relationship to the fatigue status of an athlete during a 4-week high-volume load training cycle in members of the National Rowing Team. The main finding of our study was that the distribution of HR zones and the distribution of effort-based zones were similar during the baseline week; however, the proportion of *Moderate* and *Hard* rated sessions significantly increased along with an increase in training volume, while no changes were found in the respective proportion of the HR zones.

The quantification of training sessions into different zones is a common practice in endurance disciplines (Seiler and Kjerland, 2006; Esteve-Lanao et al., 2007) to optimize performance gains and to prevent overtraining. Mostly those quantifications have been based on HR usually applied to calculate training load, however, RPE-based quantifications have also been presented (Seiler and Kjerland, 2006; Pind et al., 2019; Jesus et al., 2021). When comparing the distributions of HR and effort-based zones, there were no significant differences in the baseline week (Figure 2). It has been shown that sRPE is highly consistent with the HR measures (Foster et al., 2001). In addition, Seiler and Kjerland (2006) found no difference in intensity distribution between HR and RPE during a 32-day pre-competition preparation period in high-level cross-country skiers. They, however, distributed training bouts based on the RPE ratings, without calculating internal load (i.e., sRPE1, sRPE2, and sRPE3), thus the duration of each session was not considered. Recent studies indicate that it is not solely intensity, but also the duration of training sessions (Hofmann and Tschakert, 2017) that influence fatigue and therefore could modify post-training RPE response (Fusco et al., 2020a). Usually, differences in RPE are related to the intensity of the sessions only without including duration effects. In our study, most of the sessions were of low intensity, while a significantly higher intensity (lactate concentration about 6.0 mmol L^−1^) was used by Fusco et al. (2020a). In contrast, training at approximately 70% of VO_2max_ intensity with manipulation of exercise duration between 20 and 40 min did not significantly influence RPE response (Green et al., 2009). Very recently, it was further indicated that at lower intensities (RPE response “*Easy*”), the increase of session from 15 to 30 min did not increase post-session RPE response, while a significant increase was found for “*Moderate”* and “*Strong*” intensities in recreational runners (Jesus et al., 2021). Based on this, we can conclude that at lower intensities, as the subjects in our study were of high level with high endurance capacities, the similarities of HR and sRPE distribution during the baseline week with regular training volume could be expected due to the low intensities applied and a duration short enough not to fatigue within a single session.

During the weeks of increased training volume (week 2 and week 3), the proportion of the training sessions within the respective HR zones did not change, with approximately 20% of training performed at higher intensities than VT1. This is also consistent with what was found in the literature for endurance disciplines (Seiler and Kjerland, 2006; Seiler, 2010). Interestingly, there was a decrease in the number of *Easy* rated sessions and an increase in the *Moderate* rated sessions during week 2 and week 3 which resulted in significant differences when compared to the respective HR zones (Figure 2). Moreover, there was a tendency (*p* = 0.06) of a higher amount of sRPE3 in week 4 compared to HR Z3. Consequently, there was a shift in the RPE responses of the athletes indicating that sessions, in general, became harder. At the same time, no changes in the respective HR indices were found. First, this finding can be explained by the significant increases in session duration during weeks 3 and 4 which can cause higher acute fatigue, the concept that recent studies in the literature have also indicated (Barroso et al., 2015; Fusco et al., 2020a; Jesus et al., 2021). Jesus et al. (2021) found that using the intensities relative to RPE value 3 already caused increases in RPE if exercise duration increased from 15 to 30 min. As the subjects in our study were high-level rowers with high-performance capacities, the *Easy* session represented RPE values ≤ 4 that correspond to intensities up to an aerobic threshold (Seiler and Kjerland, 2006). Therefore, significant increases in session volume, despite being 10 min on the average for weeks 3 and 4 compared to week 1, might contribute to changes in post-exercise RPE for our subjects. Second, during such high-volume periods, training stress might increase recovery demand which disturbs the balance between training and recovery resulting in non-functional overreaching (Meeusen et al., 2013). Accordingly, we found significant increases in several stress scales because of increased training volume and, interestingly, some of them (i.e., *Social Stress, Conflicts/Pressure, Fatigue, Physical Complaints*, and *Disturbed Breaks*) even increased (*p* < 0.05) during the recovery week. The increased scores for *Fatigue* in week 3 can be expected after a 50% training volume increase in week 2 compared to the baseline week (Figure 1). Consequently, the significant increase in *Fatigue* after the recovery week (week 4) was somewhat unexpected. The reason for that is difficult to explain, but most likely the overall training load during week 4 was still too high which did not allow the dominance of recovery processes. Also, we cannot rule out the training intensity distribution effect on fatigue accumulation as Guellich et al. (2009) reported a 95.5% zone distribution of total rowing time performed at either lower or higher intensities compared to VT1. Therefore, we suggested that the overall athletic status by the end of week 4 was still compromised (Jürimäe et al., 2002; González-Boto et al., 2008; Halson, 2014) which were also reflected by higher RPE responses. Similarly, it has been indicated that accumulation of fatigue can influence RPE response after sequential days of relatively hard mixed-intensity sessions (Fusco et al., 2020b). Increased RPE scores have been found in the last stages of short (5–7 days) and long (weeks 2–3) in a 21-day cycling race, resulting in an increase of the slope of the relationship between HR and RPE TRIMP scores (Rodríguez-Marroyo et al., 2012). These studies and our current results clearly indicate that RPE might be a more sensitive parameter to calculate internal training load compared to methods based on HR time-in-zone calculations, especially during fatigue accumulation to prevent overtraining (Rietjens et al., 2005).

To the best of our knowledge, the current study is the first to investigate RPE-based training quantification during a volume increase, mainly low-intensity training cycle, and how this quantification might reflect the fatigue status. Recent studies showing the possible effect of fatigue on post-exercise RPE have rather used higher intensities (>VT2) (Fusco et al., 2020a), mixed intensities (Fusco et al., 2020b), being very short on session volume (Green et al., 2009; Jesus et al., 2021), or have used cycling tours (Rodríguez-Marroyo et al., 2012; Sanders et al., 2018). Our study focuses on a common preparatory period mesocycle where low-intensity trainings below VT1 are dominant. Additionally, an important finding of the study was that the *Fatigue* scale was related to the amount of RPE-based *Moderate* (sRPE2) and *Hard* (sRPE3) effort sessions (*p* = 0.011 and *p* = 0.008, respectively; Table 2), while no effects were found for HR-based session quantifications. Those findings indicate that quantification of training load by effort-based zones might have further advantages compared to single load (sRPE or TRIMP) quantification. A similar hypothesis was prescribed by Hofmann and Tschakert (2017) who pointed out that manipulating exercise duration at a similar training intensity results in different levels of fatigue, and therefore the stress level of athletes. Hence, training prescribed at HR < VT1, for example, may be quantified by 3 or 6, based on different durations or the fatigue status of the athletes, which in practice might change the training session effect significantly (Hofmann and Tschakert, 2017).

It has been proposed that training at higher intensities than VT1 should be well balanced for moderate and high intensities and should not exceed 20% of overall training volume to avoid excessive sympathetic stress (Seiler, 2010). The same might apply for RPE-based quantification such as a certain amount of high load can be tolerated by athletes inducing positive adaptations. This high load can either be the result of a hard and short high-intensity session or a hard rated low-intensity session if performed sufficiently for a long duration. It should also be indicated that total sRPE load (AU) during the 4 weeks was negatively associated with fatigue accumulation. This surprising finding is probably the result of the increase in fatigue level despite a significant decrease in training load at week 4. Future studies are clearly warranted to study the applicability of effort-based session quantification in the use of different training periods. Based on the current results, our proof of concept study extends the knowledge in the literature that during low-intensity, high-volume training cycles, a state of increased fatigue can be determined better by RPE rather than by HR-based methods to determine the excessive development of exercise-related stress; however, it should be the aim for the future studies to target different training periods, different disciplines, and for potential changes in performance.

The current study has some potential limitations that need to be considered. First, it was not a prospective experimental study to directly compare the effects of different session quantification methods related to either the increases in training volume or fatigue. Second, as it was not a laboratory-controlled study, different factors like hydration status or glycogen depletion (Snyder, 1998), or different HR-RPE method interaction depending on the exercise type (Lupo et al., 2016) might affect HR or RPE response to a different extent and needs to be considered. The diet of the subjects was not controlled; however, as the subjects of the study were national team candidates, they have had counseling on proper nutrition. Also, the short period of data collection (4 weeks) may be considered as a limitation of the study. In contrast, this period stands for a full mesocycle and was conducted under real field conditions having the well-trained athletes as sample. For future studies, an additional type of mesocycles needs to be investigated by applying the same proven and useful concept. Finally, we did not study training effects on performance during the 4-week period. We have recently found that different post-exercise RPE values could modify training effects (unpublished data), therefore targeting for certain RPE, based on session aim could give further information on the effect of the session on adaptation of athletes.

## Conclusion

Our study indicates that during a volume increased low-intensity training cycle in rowers, RPE-based training quantification indicated a shift toward harder rated sessions compared to unhanged HR quantification. RPE-based *Moderate* (sRPE2) and *Hard* (sRPE3) sessions were related to an increase in the RESTQ-Sport *Fatigue* scale.

## Practical Applications

The precise quantification of training load for every individual could contribute to a more accurate assessment of how the athlete is responding to the prescribed training. Frequent monitoring of parameters such as perceived exertion and fatigue during periods of volume increased training cycles may help to individualize training for proper adaptation to endurance training or preventing non-functional overreaching. To have an impact on performance, we must be sure of the nature of the relationship between the prescribed exercise dose and the expected training outcome or response. The HR-based time-in-zone approach is rather an easy method to measure training load; however, the HR might drift to lower values over the course of a longer workout or training cycles. To the best of our knowledge, the current study is the first to investigate RPE-based training quantification during a volume increased, mainly low-intensity training cycle. RPE drifted toward harder rated sessions compared to unchanged HR quantification, which was supported by the RESTQ-Sport *Fatigue* scale. Therefore, RPE may be particularly more useful compared to the HR-based methods to determine the excessive development of exercise stress in well-trained rowers or endurance athletes in general.

## Data Availability Statement

The original contributions presented in the study are included in the article/supplementary material, further inquiries can be directed to the corresponding author.

## Ethics Statement

The studies involving human participants were reviewed and approved by Declaration of Helsinki and was approved by the Institutional Ethical Committee (University of Tartu). Written informed consent to participate in this study was provided by the participants' legal guardian/next of kin.

## Author Contributions

All authors contributed to the data analysis and interpretation of the data, drafting and revising the manuscript, and approved the final version of the manuscript.

## Funding

This study was supported by the Estonian Research Council Grant No. PUT1395G.

## Conflict of Interest

The authors declare that the research was conducted in the absence of any commercial or financial relationships that could be construed as a potential conflict of interest.

## Publisher's Note

All claims expressed in this article are solely those of the authors and do not necessarily represent those of their affiliated organizations, or those of the publisher, the editors and the reviewers. Any product that may be evaluated in this article, or claim that may be made by its manufacturer, is not guaranteed or endorsed by the publisher.
