# Corrigendum: Increases in RPE Rating Predict Fatigue Accumulation Without Changes in Heart Rate Zone Distribution After 4-Week Low-Intensity High-Volume Training Period in High-Level Rowers

**DOI:** 10.3389/fphys.2022.834667

**Published:** 2022-01-31

**Authors:** Rasmus Pind, Peter Hofmann, Evelin Mäestu, Eno Vahtra, Priit Purge, Jarek Mäestu

**Affiliations:** ^1^Institute of Sport Sciences and Physiotherapy, University of Tartu, Tartu, Estonia; ^2^Institute of Human Movement Science, Sport and Health, University of Graz, Graz, Austria

**Keywords:** training monitoring, intensity, duration, internal load, external load, exercise prescription, session RPE

In the original article, there was a mistake in Table 1 as published. Some average values in Week 4 column (those with no significant change) have been lost and only standard deviations appear. The corrected Table 1 appears below.

**Table 1:** Average scores (mean ± SD) of the Recovery-Stress Questionnaire for Athletes (RESTQ-Sport) scales during the 4-week training period.

**Scale**	**Baseline week**	**Week 2**	**Week 3**	**Week 4**	***P*-value**
**General stress**					
1. General stress	1.01 ± 0.93	1.03 ± 1.08	1.25 ± 1.03	1.97 ± 1.68	ns
2. Emotional stress	1.21 ± 0.90	1.13 ± 1.10	1.28 ± 0.88	2.09 ± 1.58	ns
3. Social stress	1.29 ± 0.82	1.11 ± 0.94	1.33 ± 0.91	**2.25** **± 1.38**^**b**^	*p* < 0.05
4. Conflicts/pressure	1.65 ± 0.90	1.61 ± 0.87	1.93 ± 0.71	**2.59** **± 1.19**^**b**^	*p* < 0.05
5. Fatigue	1.74 ± 1.19	1.61 ± 1.11	**2.07** **± 1.23**^**b**^	**2.69** **± 1.50**^**b**^	*p* < 0.05
6. Lack of energy	1.81 ± 0.82	1.53 ± 0.69	1.70 ± 0.73	1.75 ± 0.53	ns
7. Physical complaints	1.44 ± 0.75	1.52 ± 0.83	1.68 ± 0.64	**1.91** **± 0.58**^**a**^	*p* < 0.05
**General recovery**					
8. Success	3.11 ± 0.79	2.75 ± 1.25	2.55 ± 0.92	2.75 ± 0.88	ns
9. Social recovery	3.57 ± 1.36	3.42 ± 1.35	3.47 ± 1.31	2.50 ± 1.20	ns
10. Physical recovery	3.18 ± 1.18	3.08 ± 1.08	2.83 ± 1.07	2.34 ± 1.18	ns
11. General well-being	3.96 ± 1.32	3.94 ± 1.31	3.93 ± 1.27	**2.69** **± 1.27**^**a**^	*p* < 0.05
12. Sleep quality	4.00 ± 1.08	**4.53** **± 0.90**^**a**^	4.15 ± 1.13	3.31 ± 1.46	*p* < 0.05
**Sport stress**					
13. Disturbed breaks	1.47 ± 0.82	1.09 ± 0.50	**1.63** **± 0.93**^**b**^	**1.94** **± 0.94**^**b**^	*p* < 0.05
14. Emotional exhaustion	1.07 ± 1.36	1.03 ± 1.14	1.30 ± 1.31	1.69 ± 1.93	ns
15. Injury	2.13 ± 0.98	2.19 ± 1.03	**2.03** **± 0.92**^**b**^	1.63 ± 0.82	*p* < 0.05
**Sport recovery**					
16. Being in shape	3.51 ± 1.11	3.39 ± 1.24	3.00 ± 1.09	2.84 ± 0.88	ns
17. Personal accomplishments	3.19 ± 0.99	2.88 ± 1.14	**2.50** **± 1.29**^a^	**2.22** **± 1.48**^a^	*p* < 0.05
18. Self-efficacy	3.44 ± 0.76	3.41 ± 0.98	3.03 ± 1.00	3.25 ± 0.96	ns
19. Self-regulation	2.31 ± 0.83	2.47 ± 0.88	2.37 ± 0.94	**3.44** **± 1.24**^**b**^	*p* < 0.05

a *p < 0.05 is significantly different from baseline week*.

b *p < 0.05 is significantly different from week 2. ns, non-significant. The statistical differences (p < 0.05) are shown in bold*.

The authors apologize for this error and state that this does not change the scientific conclusions of the article in any way. The original article has been updated.

## Publisher's Note

All claims expressed in this article are solely those of the authors and do not necessarily represent those of their affiliated organizations, or those of the publisher, the editors and the reviewers. Any product that may be evaluated in this article, or claim that may be made by its manufacturer, is not guaranteed or endorsed by the publisher.

